# Quantifying the Multiscale Predictability of Financial Time Series by an Information-Theoretic Approach

**DOI:** 10.3390/e21070684

**Published:** 2019-07-12

**Authors:** Xiaojun Zhao, Chenxu Liang, Na Zhang, Pengjian Shang

**Affiliations:** 1 School of Economics and Management, Beijing Jiaotong University, Beijing 100044, China; 2 School of Science, Beijing Jiaotong University, Beijing 100044, China

**Keywords:** predictability, multiscale analysis, entropy rate, memory effect, financial time series

## Abstract

Making predictions on the dynamics of time series of a system is a very interesting topic. A fundamental prerequisite of this work is to evaluate the predictability of the system over a wide range of time. In this paper, we propose an information-theoretic tool, multiscale entropy difference (MED), to evaluate the predictability of nonlinear financial time series on multiple time scales. We discuss the predictability of the isolated system and open systems, respectively. Evidence from the analysis of the logistic map, Hénon map, and the Lorenz system manifests that the MED method is accurate, robust, and has a wide range of applications. We apply the new method to five-minute high-frequency data and the daily data of Chinese stock markets. Results show that the logarithmic change of stock price (logarithmic return) has a lower possibility of being predicted than the volatility. The logarithmic change of trading volume contributes significantly to the prediction of the logarithmic change of stock price on multiple time scales. The daily data are found to have a larger possibility of being predicted than the five-minute high-frequency data. This indicates that the arbitrage opportunity exists in the Chinese stock markets, which thus cannot be approximated by the effective market hypothesis (EMH).

## 1. Introduction

Making predictions on the dynamics of time series of a system is a very interesting topic. Up to now, over thousands of methods have been proposed for the prediction of the systems’ evolution [1]. A fundamental prerequisite of these works is to evaluate the predictability of the system over a wide range of time. For an isolated system, which does not exchange information with other systems, the predictability of the output time series is only determined by the degree of memory from the past values. In such a case, the time series in unpredictable if it is purely random, like Gaussian white noise; whereas, information can be extracted for prediction by analyzing the temporal structure of a time series with memory. In another way, examples of irreversible processes include typically chaotic dissipative processes, nonlinear stochastic processes, and processes with memory, operating away from thermodynamic equilibrium. One should be able to make easier predictions on irreversible processes, where the arrow of time is playing a role, than on reversible ones [2,3]. For a real-world system that may exchange information with other systems, the past values of other systems can also be utilized for prediction, except the past values of the underlying system itself [4,5].

In time series analysis, the multiscale analysis of time series has been broadly studied, which relies on the fact that the time series of complex systems, associated with a hierarchy of interacting regulatory mechanisms, usually generate complex fluctuations over multiple time scales. Analyzing the financial time series by amplification in different proportions with a coarse-graining algorithm [6] makes it possible to reveal both small-scale information and large-scale information at multiple resolutions. This paper contributes to evaluating the multiscale predictability of financial time series. Another piece of evidence of this consideration is that the multiscale complexity (a tool of time series analysis that is associated with factors of the degree of memory, the temporal structure, and auto-correlations) have been measured [6,7], and hence, the predictability of time series, which is also closely related to those factors, can be analyzed on multiple time scales as well.

Financial time series analyses have played an important role in developing some of the fundamental economic theories. Furthermore, the understanding and analysis of financial time series, especially the evolution of stock markets, has been attracting the close attention of economists, statisticians, and mathematicians for many decades [8,9,10,11,12,13,14]. Recent research mostly focuses on the long-term average behavior of a market, and thus sheds little light on the temporal changes of a market. This type of method for analyzing financial time series may lead to a lack of analysis on the short-term predictability of time series, thus ignoring the critical information that is contained in the financial data, which may be used for the portfolio selection and pursuing an arbitrage opportunity [15].

If the efficient market hypothesis (EMH) is of some relevance to reality, then a market would be very unpredictable due to the possibility for investors to digest any new information instantly [16]. When a market behaves as the EMH stipulates, the market will be purely random without memory, and the variation of price will be very unpredictable. For an extensive review of the EMH, please see [17]. However, new evidence challenges the EMH with many empirical facts from observations, e.g., the leptokurtosis and fat tail of the non-Gaussian distribution, especially the fractal market hypothesis (FMH) [18]. The FMH asserts that (i) a market consists of many investors with different investment horizons, and (ii) the information set that is important to each investment horizon is different. As long as the market maintains this fractal structure, with no characteristic time scale, the market remains stable. When the market’s investment horizon becomes uniform, the market becomes unstable because everyone is trading based on the same information set. In addition, Beben and Orlowski [19] and Di Matteo et al. [20,21] found that emerging markets were likely to have a stronger degree of memory than developed markets, suggesting that the emerging markets had a larger possibility of being predicted.

In this paper, we incorporate the multiscale analysis with an information-theoretic approach for characterizing the degree of memory of time series, so as to evaluate the predication of financial time series. We make use of the entropy rate in order to test the predictability of some synthetic data and of the Chinese stock markets. It is an interesting alternative to regression models, which are often used in financial time series. One advantage is that the method proposed is mainly model independent; another is that it deals with nonlinear systems, as well as with linear ones. The remainder of the paper is organized as follows. In the Methodology Section, we introduce a new entropy difference (ED) and its multiscale case, multiscale entropy difference (MED). We then apply these new methods to the numerical analysis of artificial simulations, including the logistic map, the Hénon map, the Lorenz system, and most importantly, the financial time series analysis. Finally, we give a brief conclusion.

## 2. Methodology

### 2.1. Entropy Difference

(i) For an isolated system, which does not exchange information with other systems, the degree of predictability of the time series can only be explained by the memory effects of its past values.

As the output of the underlying system, a time series $\left\{x_{t}\right\}$, t=1,⋯,T is considered. First, the uncertainty of the time series at time *t* can be quantified by the Shannon entropy:

(1) $$H\left[x_{t}\right]=\sum_{x_{t}\in \Theta }p\left(x_{t}\right)log_{2}p\left(x_{t}\right).$$

$p(x_{t})$ represents the probability distribution of $x_{t}$; Θ is the space of samples; and $H[x_{t}]$ describes the information of *x* at time *t* in bits.

The entropy rate measures the net information generated by the system at time *t*, given by $H[x_{t}|x_{1},x_{2},\cdots ,x_{t-1}]$. We assume that the underlying system can be approximated by a *p*-order Markov process. That is to say, the value of the output time series at time *t* is only related to its nearest *p* neighbors and is independent of further values. Therefore, we obtain $H\left[x_{t}|x_{1},x_{2},\cdots ,x_{t-1}\right]=H\left[x_{t}|x_{t-p},x_{t-p+1},\cdots ,x_{t-1}\right]\equiv H\left[x_{t}|x_{t-1}^{\left(p\right)}\right]$, where:

(2) $$H\left[x_{t}|x_{t-1}^{\left(p\right)}\right]=\sum_{x_{t},x_{t-1}^{\left(p\right)}\in \Theta }p\left(x_{t},x_{t-1}^{\left(p\right)}\right)log_{2}\frac{p(x_{t},x_{t-1}^{\left(p\right)})}{p(x_{t-1}^{\left(p\right)})}.$$

The uncertainty of the time series at time *t* is non-increasing given the past values, and hence, the entropy rate is no larger than the entropy itself: $H\left[x_{t}|x_{t-1}^{\left(p\right)}\right]\leq H\left[x_{t}\right]$.

The difference between the Shannon entropy and the entropy rate represents the contributions of the past values to reducing the uncertainty (and improving the predictability) of the time series at time *t*. It is given by:

(3) $$D=H\left[x_{t}\right]-H\left[x_{t}|x_{t-1}^{\left(p\right)}\right].$$

We name *D* the entropy difference (ED). For any (nonlinear) time series, D≥0. For a random walk process, the contribution of past values is negligible; hence, $H\left[x_{t}|x_{t-1}^{\left(p\right)}\right]=H\left[x_{t}\right]$, and $H\left[x_{t}\right]-H\left[x_{t}|x_{t-1}^{\left(p\right)}\right]=0$. *D* equal to zero indicates that the time series cannot be predicted at all, as no past information can be utilized; whereas, if there exist autocorrelations/memory effects within the time series, the past values can be used to reduce the uncertainty of time series at time *t*, so D>0.

The entropy difference *D* is non-negative, while the upper bound of *D* is uncertain. Thus, we further normalize *D* to the range of [0,1], divided by its maximum value $H[x_{t}]$:

(4) $$D=\frac{H\left[x_{t}\right]-H\left[x_{t}|x_{t-1}^{\left(p\right)}\right]}{H[x_{t}]}=1-\frac{H[x_{t}|x_{t-1}^{\left(p\right)}]}{H[x_{t}]}.$$

Here, 0≤D≤1. The normalized ED, D, quantifies the degree of predictability of the time series. Similarly, when D is approximately 0, the time series is unpredictable. When D attains a value of one, $H[x_{t}|x_{t-1}^{\left(p\right)}]$ is approximately 0. Therefore, there exists no uncertainty of $x_{t}$ in the presence of the past values $x_{t-1}^{\left(p\right)}$, and the time series is completely specified (well predicted) at time *t*.

(ii) Next, consider a real-world system that exchanges information with other systems. Except the past values of the underlying system itself, the past values of other systems can also be exploited. Revisit the Granger causality, which is a statistical concept of causality that is based on prediction [22,23]. If a signal *y* “Granger-causes” a signal *x*, then past values of *y* should contain information that helps predict *y* above and beyond the information contained in past values of *x* alone. In the Granger causality, the value of $x_{t}$ is predicted by two equations, respectively,

(5) $$\begin{array}{cc} x_{t} & =\sum_{i=1}^{p}\alpha_{i}x_{t-i}+\epsilon_{1t}.\\ x_{t} & =\sum_{i=1}^{p}\beta_{i}x_{t-i}+\sum_{j=1}^{q}\gamma_{j}y_{t-j}+\epsilon_{2t}. \end{array}$$

The Granger causality is normally tested in the context of linear regression models. If the second forecast is found to be more successful, according to standard cost functions, then the past of *y* appears to contain information helping in forecasting $x_{t}$ that is not in past $x_{t-1}^{\left(p\right)}$. The Akaike information criterion (AIC) or Bayesian information criterion (BIC) can be adopted to determine the lagged ranks *p* and *q*. The residual terms $\epsilon_{1t}$ and $\epsilon_{2t}$, as a matter of fact, contain the information generated by the system at time *t*. A nonlinear extension of the Granger causality is the information-theoretic tool of transfer entropy [24,25], which measures the information flow from *y* to *x*:

(6) $$\begin{array}{cc} T_{y\to x} & =H\left[x_{t}|x_{t-1}^{\left(p\right)}\right]-H\left[x_{t}|x_{t-1}^{\left(p\right)},y_{t-1}^{\left(q\right)}\right]\\ & =\sum_{\begin{array}{c} x_{t},x_{t-1}^{\left(q\right)}\in \Theta \\ y_{t-1}^{\left(q\right)}\in \Xi \end{array}}p\left(x_{t},x_{t-1}^{\left(p\right)},y_{t-1}^{\left(q\right)}\right)log_{2}\frac{p(x_{t}|x_{t-1}^{\left(p\right)},y_{t-1}^{\left(q\right)})}{p(x_{t}|x_{t-1}^{\left(p\right)})}. \end{array}$$

Both the Granger causality and the transfer entropy indicate that the past values of another related system can be used to infer the trajectory of the underlying system. Hence, the ED of the isolated system can be extended to the multiple systems case.

The entropy rate of one system in the presence of another coupled system is given by $H[x_{t}|x_{t-1}^{\left(t-1\right)},y_{t-1}^{\left(t-1\right)}]$. We further assume that these two systems can be approximated by the generalized Markov processes [24], that is $H\left[x_{t}|x_{t-1}^{\left(t-1\right)},y_{t-1}^{\left(t-1\right)}\right]=H\left[x_{t}|x_{t-1}^{\left(p\right)},y_{t-1}^{\left(q\right)}\right]$, and:

(7) $$\begin{array}{c} H\left[x_{t}|x_{t-1}^{\left(p\right)},y_{t-1}^{\left(q\right)}\right]=H\left[x_{t},x_{t-1}^{\left(p\right)},y_{t-1}^{\left(q\right)}\right]-H\left[x_{t-1}^{\left(p\right)},y_{t-1}^{\left(q\right)}\right]\\ =\sum_{\begin{array}{c} x_{t},x_{t-1}^{\left(q\right)}\in \Theta \\ y_{t-1}^{\left(q\right)}\in \Xi \end{array}}p\left(x_{t},x_{t-1}^{\left(p\right)},y_{t-1}^{\left(q\right)}\right)log_{2}\frac{p(x_{t},x_{t-1}^{\left(p\right)},y_{t-1}^{\left(q\right)})}{p(x_{t-1}^{\left(p\right)},y_{t-1}^{\left(q\right)})}. \end{array}$$

The uncertainty of system *x* can be given by the conditional probability distribution $p(x_{t}|x_{t-1}^{\left(p\right)},y_{t-1}^{\left(q\right)})$. The conditional probability distribution $p(x_{t}|x_{t-1}^{\left(p\right)},y_{t-1}^{\left(q\right)})$ describes the data range and the occurrence probability of $x_{t}$ by knowing the past values of $x_{t-1}^{\left(p\right)},y_{t-1}^{\left(q\right)}$. Consider an extreme case. If $p(x_{t}\equiv c|x_{t-1}^{\left(p\right)},y_{t-1}^{\left(q\right)})$, where *c* is a constant, then $x_{t}$ is fixed at point *c* with no uncertainty. Further, when the conditional distribution is fixed within a narrow range, the system is more deterministic at time *t* by knowing $x_{t-1}^{\left(p\right)},y_{t-1}^{\left(q\right)}$, which can thus be well predicted. If the conditional distribution is still wide in the range, the system is full of uncertainty at time *t* and has a low possibility of being predicted.

The reduced uncertainty by knowing the past values of both *x* and *y* is estimated by the ED:

(8) $$D=H\left[x_{t}\right]-H\left[x_{t}|x_{t-1}^{\left(p\right)},y_{t-1}^{\left(q\right)}\right].$$

Further, the ED is normalized by:

(9) $$D=\frac{H\left[x_{t}\right]-H\left[x_{t}|x_{t-1}^{\left(p\right)},y_{t-1}^{\left(q\right)}\right]}{H[x_{t}]}=1-\frac{H[x_{t}|x_{t-1}^{\left(p\right)},y_{t-1}^{\left(q\right)}]}{H[x_{t}]}.$$

D ranges between 0 and 1. D being approximately 0 indicates a low degree of predictability of the time series, and D close to 1 indicates a large degree of predictability. In addition, to set the ED in a fixed range, the normalization of ED also has other merits. Below is the explanation.

The predictability of a system is mainly subjected to the contributions of two aspects:

(i) The degree of the memory of the underlying system, that the past information can be well utilized to infer the future evolution of the system;
(ii) Whether a system is more deterministic than other systems. This is related to the range of the fluctuations of the time series, which can be partly explained by the variance of the time series. A time series with large variance (entropy) tends to be more difficult to predict than a time series with much small variance. Both the variance and the entropy reflect the diversity of the system. A system with more diverse states is likely to have large variance and entropy, whereas a system with few states tends to have small ones. Obviously, a system with fewer states is easier to predict than that with diverse states.

Therefore, the normalization of ED by dividing *D* by $H[x_{t}]$ makes it possible to compare the degree of predictability between different systems, even if they have different ranges of fluctuations. Moreover, regarding the estimation of entropy values from time series, there may exist biases for different estimators. The normalization can offset those biases caused by the estimation of entropy if the numerator and the denominator use the same estimator.

Further, for a more complicated case of multiple subsystems (larger than 2 subsystems), e.g., the Lorenz system, the predictability of the time series can be given by:

(10) $$\begin{array}{cc} D & =\frac{H\left[x_{t}\right]-H\left[x_{t}|x_{t-1}^{\left(p\right)},y_{t-1}^{\left(q\right)},z_{t-1}^{\left(l\right)}\right]}{H[x_{t}]}\\ & =1-\frac{H[x_{t}|x_{t-1}^{\left(p\right)},y_{t-1}^{\left(q\right)},z_{t-1}^{\left(l\right)}]}{H[x_{t}]}, \end{array}$$

when the past values of *x*, *y*, and *z* can be used to predict $x_{t}$. Here, $Z_{t-1}^{\left(l\right)}$ could be a vector of possible explanatory variables.

### 2.2. Multiscale Entropy Difference

The predictability of time series estimated by ED and the normalized version is given on a unique time scale, on which the data are sampled. Here, we further evaluate that the multiscale predictability of time series relies on the fact that the time series of complex systems, associated with a hierarchy of interacting regulatory mechanisms, usually generate complex fluctuations over multiple time scales. There exist many approaches for the multiscale analysis in the framework of fractal theory [26], e.g., the data segments of detrended fluctuation analysis (DFA) [27], coarse-graining [6], and the time delay of phase space reconstruction [28,29], where the coarse-graining is one of the simplest methods.

We coarse grained the original data onto multiple time scales with a scale parameter *s* [2,6,7]. By the non-overlapping coarse-graining, the original time series *x* (with length *T*) is rescaled to X(s):

(11) $$X_{t}\left(s\right)=\frac{1}{s}\sum_{k=(t-1)s+1}^{ts}x_{k}.$$

*t* ranges from 1 to T/s. $X_{t}\left(s\right)$ represents the moving average of the system *x* at time *t* on the temporal scale *s*. The coarse-graining process is a low-pass filter, where the high-frequency fluctuations are filtered out. At small time scales, the details of the time series can be reserved, while at large scales, the details are ignored and only the profile of the time series is retained.

The procedure of the multiscale entropy difference (MED) mainly includes 3 steps:

Step 1. Coarse grain the original time series $\left\{x_{t}\right\}$ (t=1,⋯,T) to the coarse-grained time series $\left\{X_{t}\left(s\right)\right\}$ (t=1,⋯,T/s), with a time scale *s*.

Step 2. Estimate the ED and the normalized ED for the coarse-grained time series $\left\{X_{t}\left(s\right)\right\}$ (t=1,⋯,T/s), respectively.

Step 3. Change the time scale *s* and observe the changes of ED, and the normalized ED, on different time scales. 

When the scale *s* is equal to 1, the MED method retrieves back the ED method. For other scales, the MED can evaluate the multiscale predictability of the time series. To be noted, for a short time series of length *T*, the multiscale analysis may be affected by the finite size effects at large time scales, which can be solved by the refined entropy estimators during the coarse-graining process. For more details, please see [5,30,31].

## 3. Numerical Simulations

In this section, we consider three examples to test our new methods, including one isolated system and two open systems.

We first consider the logistic map. It is a polynomial mapping of degree two, which consists of only one nonlinear system: $x_{t}=\mu x_{t-1}\left(1-x_{t-1}\right)$. For ∀t, $x_{t}\in \left[0,1\right]$ can be used to represent the ratio of existing population to the maximum possible population in ecology. The values of interest for the parameter μ are those in the interval [0, 4]. Complex, chaotic behavior can arise from this very simple non-linear dynamical equation. Most values of μ beyond 3.56995 exhibit chaotic behavior. Here, we set μ=3.7 and let the data length $T=10^{5}$. The initial value of $x_{0}$ was set to 0.5.

As only one equation is described in the logistic map, $x_{t}$ changes no information with other variables. We added Gaussian white noises on the original time series $x_{t}$ with different strengths to obtain a composite time series: $y_{t}=x_{t}+\lambda \epsilon_{t}$. $\epsilon_{t}$ is the Gaussian white noise (with zero mean and unit variance). λ≥0 is a parameter that tunes the strength of noises. $x_{t}$ is the real signal corrupted by the external noise $\epsilon_{t}$, and λ determines the signal-noise ratio. The larger λ, the smaller the signal-noise ratio is.

**Figure 1:**
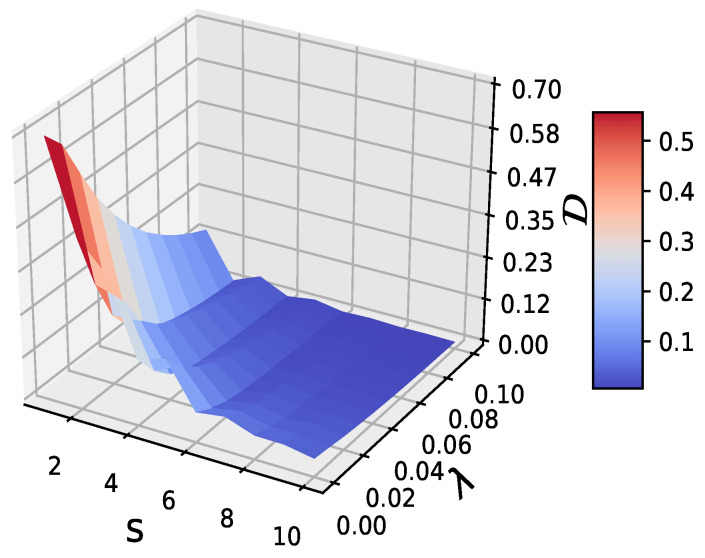
The values of D (Equation 4, lagged rank p=1) on multiple time scales s=1,2,⋯,10, with the noise strength parameter λ=0.01,0.02,⋯,0.1 for the logistic map.

We used *k*-means clustering [32] to discretize the original data into *k* symbols, so as to estimate the entropies. *k* is a pre-defined parameter that determines the number of clusterings. Here, the parameter *k* for the *k*-means clustering was 10, i.e., we symbolized the original continuous time series as 10 discrete symbols. In Figure 1, we show the values of normalized EDD on multiple time scales s=1,2,⋯,10, with the noise strength parameter λ from 0.01 to 0.1 with a step of 0.01, since the variance of the original time series *x* of the logistic map ($T=10^{5})$ was only 0.0412, the original data length was $T=10^{5}$, therefore, even at s=10, this ensured that the coarse-grained data length was $10^{4}$. For s=1 and λ=0, corresponding to the original time series *x*, the degree of predictability was larger than 0.7. This indicates that the logistic map had a large possibility of being predicted, which coincides well with what the equation describes. When the scale increased, the predictability of the coarse-grained time series decreased, since the relationship between $X_{t}\left(s\right)$ and $X_{t-1}\left(s\right)$ became weaker on large scales. Moreover, the predictability of the time series also decreased with increasing λ, as the signal-noise ratio became lower. D reached a value very close to zero when λ=0.1, so the composite time series could not be predicted. We also tested other values of *k*, for which it turned out that the values of larger *k* gave more reliable results; however, this was limited by the original data length. We further generated several groups of Gaussian white noises to add on the original time series and obtained very similar results, which verified the robustness of our new methods.

Next, we considered the Hénon map, which consists of two subsystems: $x_{t}=1-ax_{t-1}^{2}+y_{t-1}$ and $y_{t}=bx_{t-1}$. The map depends on two parameters, *a* and *b*. For the classical Hénon map, it has values of a=1.4 and b=0.3. There exists nonlinear information flow from $x_{t-1}$ and $y_{t-1}$ to $x_{t}$, i.e., a one-step transition from the past data of one variable *y* to the current the data of another variable *x*. The initial values were set to (1,0).

**Figure 2:**
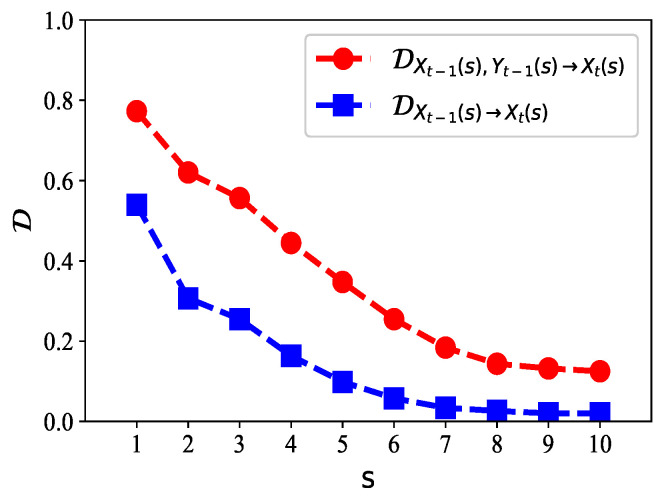
The values of D on multiple time scales s=1,2,⋯,10. The parameter *k* for the *k*-means clustering is 20. We compare $D_{X_{t-1}\left(s\right),Y_{t-1}\left(s\right)\to X_{t}\left(s\right)}$ with $D_{X_{t-1}\left(s\right)\to X_{t}\left(s\right)}$ and find that $D_{X_{t-1}\left(s\right)\to X_{t}\left(s\right)}$ is always smaller than $D_{X_{t-1}\left(s\right),Y_{t-1}\left(s\right)\to X_{t}\left(s\right)}$ on each time scale. This indicates that the predictability of *x* can be improved by incorporating the past values of *y* more than the past values of *x* alone. Therefore, the past values of *y* contain information for predicting *x*, which coincides well with the equations of the map.

We generated data with the classical Hénon map, with the data length $T=10^{5}$. In Figure 2, we show the values of normalized EDD on multiple time scales s=1,2,⋯,10. For s=1, which corresponds to the original time series *x* and *y*, the degree of predictability was 0.77. This indicates that $x_{t}$ can be well predicted by using the past values of *x* and *y*. When the scale increased, the predictability of the coarse-grained time series decreased, as the relationship among $X_{t}\left(s\right)$, $X_{t-1}\left(s\right)$, and $Y_{t-1}\left(s\right)$ became weaker on large scales. We also compare $D_{X_{t-1}\left(s\right),Y_{t-1}\left(s\right)\to X_{t}\left(s\right)}=1-H\left[X_{t}\left(s\right)|X_{t-1}\left(s\right),Y_{t-1}\left(s\right)\right]/H\left[X_{t}\left(s\right)\right]$ with $D_{X_{t-1}\left(s\right)\to X_{t}\left(s\right)}=1-H\left[X_{t}\left(s\right)|X_{t-1}\left(s\right)\right]/H\left[X_{t}\left(s\right)\right]$ in Figure 2. Here, the lagged ranks *p* and *q* were both set to one. Obviously, if we only used the past values of *x* to predict $x_{t}$, the predictability of the time series would be much lower than if we incorporated both the past values of *x* and *y*. Therefore, we always obtained $D_{X_{t-1}\left(s\right),Y_{t-1}\left(s\right)\to X_{t}\left(s\right)}\geq D_{X_{t-1}\left(s\right)\to X_{t}\left(s\right)}$. Actually, $D_{X_{t-1}\left(s\right),Y_{t-1}\left(s\right)\to X_{t}\left(s\right)}-D_{X_{t-1}\left(s\right)\to X_{t}\left(s\right)}$ is just the normalized multiscale transfer entropy [5], and its unique scale case $D_{x_{t-1},y_{t-1}\to x_{t}}-D_{x_{t-1}\to x_{t}}$ is the normalized transfer entropy [24,33], from *y* to *x*.

Third, we studied the Lorenz system [34], which consists of three subsystems: dx/dt=σ(y−x), dy/dt=x(r−z)−y, and dz/dt=xy−bz. Here, *x*, *y*, and *z* make up the system states, *t* time, and σ, *r*, and *b* the parameters: σ=10, r=28, b=8/3. We integrated these equations numerically, applying a fourth-order Runge–Kutta method with the initial values of (0.1,0,0).

**Figure 3:**
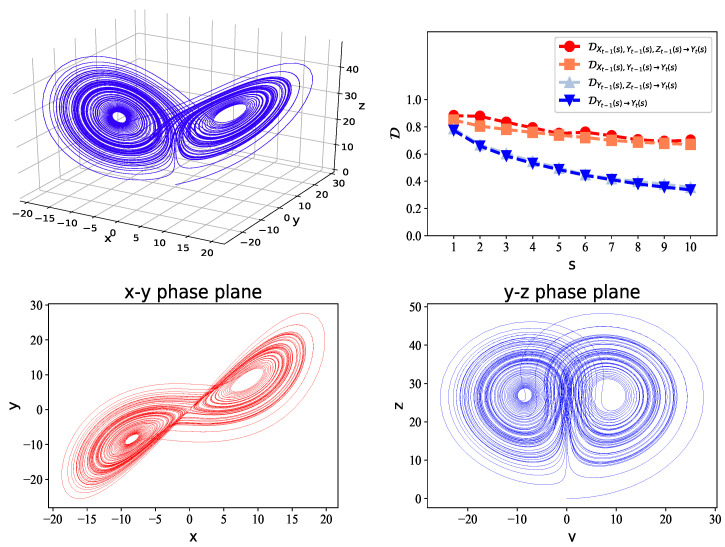
Upper left panel: A sample solution in the Lorenz system when σ=10, r=28, and b=8/3, with initial values (0.1,0,0). The data length is $T=10^{5}$. Upper right panel: the values of D on multiple time scales s=1,2,⋯,10. The parameter *k* for the *k*-means clustering is 20. Lower left panel: the *x*–*y* phase plane. Lower right panel: the *y*–*z* phase plane.

We used the Lorenz system to generate data of length $T=10^{5}$. In Figure 3, we give the values of normalized ED on multiple time scales s=1,2,⋯,10. For s=1, which corresponds to the original time series *x*, *y*, and *z*, the degree of predictability of $y_{t}$ reached 0.88. This indicates that $y_{t}$ can be well predicted by using the past values of *x*, *y*, and *z*. When the scale increased, the predictability of the coarse-grained time series decreased, as the relationship among $Y_{t}\left(s\right)$, $X_{t-1}\left(s\right)$, $Y_{t-1}\left(s\right)$ and $Z_{t-1}\left(s\right)$ became weaker on large scales. We also compared $D_{X_{t-1}\left(s\right),Y_{t-1}\left(s\right),Z_{t-1}\left(s\right)\to Y_{t}\left(s\right)}$ with $D_{X_{t-1}\left(s\right),Y_{t-1}\left(s\right)\to Y_{t}\left(s\right)}$, $D_{Y_{t-1}\left(s\right),Z_{t-1}\left(s\right)\to Y_{t}\left(s\right)}$, and $D_{Y_{t-1}\left(s\right)\to Y_{t}\left(s\right)}$. Here, the lagged ranks *p*, *q*, and *l* were all set to one. We found that $y_{t}$ can be well predicted giving the past values of *x* and *y*. Interestingly, the past values of *z* contributed much less to predicting *y*, although in the second equation of the Lorenz system, the change of *y* (dy/dt) is also explained by *z*. This can be explained as follows. In the *x*–*y* phase plane, *x* is closely related to *y* in the “diagonal” direction, as shown in Figure 3. However, in the *y*–*z* phase plane, no obvious relationship appears between *y* and *z*. Therefore, both the past values of *x* and *y* contribute to predicting *y*, rather than *z*. To predict other variables like *x* and *z*, we obtained very similar results.

## 4. Financial Time Series Analysis

The emerging stock markets have been found to have memory with the past values [35]; thus, the stock prices are not purely random. Past values can be used for the prediction of future stock prices. In this section, we study the predictability of the stock data of Shanghai and Shenzhen stock markets in China. We analyze the Shanghai Composite Index (SSE) and Shenzhen Composite Index (SZSE), both including the trading price and trading volume. At time *t*, the data related to trading price are given by $x_{t}$, and the data related to trading volume are given by $y_{t}$. Except the original data, we also analyzed the logarithmic change of stock price (i.e., logarithmic return): $log\left(x_{t}\right)-log\left(x_{t-1}\right)$, the logarithmic change of trading volume: $log\left(y_{t}\right)-log\left(y_{t-1}\right)$, the volatility (absolute return) of stock price: $|log\left(x_{t}\right)-log\left(x_{t-1}\right)|$, and the volatility of trading volume: $|log\left(y_{t}\right)-log\left(y_{t-1}\right)|$, respectively.

### 4.1. Five-Minute High-Frequency Data Analysis

**Figure 4:**
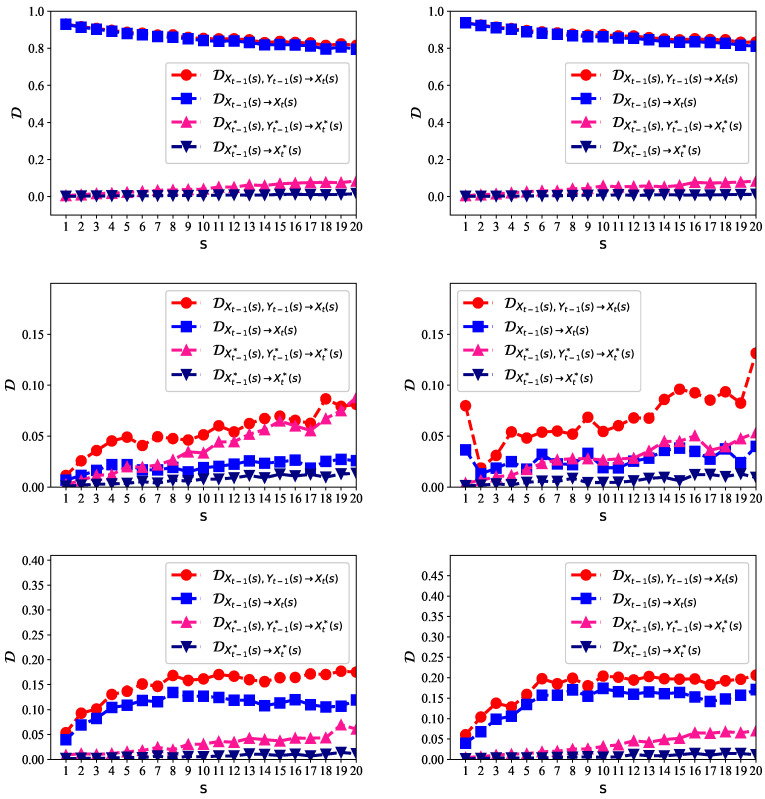
The multiscale entropy difference (MED) for the five-minute high-frequency stock data for the Shanghai and Shenzhen markets. Left panels show the results of the stock price (upper left), logarithmic return (middle left), and price volatility (lower left) for the Shanghai Composite Index (SSE), respectively. Right panels are those for the Shenzhen Composite Index (SZSE), respectively. The data related to the trading price are given by *X*, and the data related to trading volume are given by *Y*. $X^{*}$ and $Y^{*}$ represent the shuffled data.

We first analyzed the predictability of five-minute high-frequency data of SSE and SZSE. The data ranged from 3 March 2016 to 9 October 2018. In Figure 4, the left panels show the predictability of the stock price, logarithmic return, and price volatility for SSE, respectively. The right panels show the predictability of the stock price, logarithmic return, and price volatility for SZSE, respectively.

For the original non-stationary stock prices, the predictability was very high on multiple time scales (as shown on the left panels of Figure 4), with D larger than 0.8, in the presence of either $X_{t-1}$ alone or $X_{t-1}\&Y_{t-1}$. The reason is that we can just use $X_{t-1}$ as the predicted value of $X_{t}$. The prediction error would be very small, because neighboring stock prices are very close. This explains why D was so large, but such a prediction is meaningless for the arbitrage. What makes investors more interested are the logarithmic return, which indicates the price going up or down, and the price volatility, which is the indicator of risk.

On the middle panels of Figure 4, the logarithmic return is more likely to be predicted given the past values of logarithmic return and the logarithmic change of trading volume than given the past values of logarithmic return alone, that is $D_{X_{t-1}\left(s\right),Y_{t-1}\left(s\right)\to X_{t-1}\left(s\right)}>D_{X_{t-1}\left(s\right)\to X_{t-1}\left(s\right)}$. This indicates that the trading volume contributes significantly to the prediction of the stock price. The close relationship between the stock price and trading volume was also found in previous studies, e.g., [36]. We shuffled the underlying data, represented by $X^{*}$ and $Y^{*}$. The predictability for the shuffled data became much lower, because the shuffling process broke the memory among neighboring values for prediction, although it retained the distribution of the data.

We also show the results of price volatility on the lower panels of Figure 4. The degree of the predictability became larger than that of the logarithmic return. There existed long-range persistent correlations in the volatility series [37], so that the clustering of extreme volatilities emerged. A larger volatility was more likely to be followed by a large volatility, and vice versa [38,39]. The clustering of extreme volatilities made it possible to predict the volatility series from the neighboring past values. We found that the trading volume volatility can also help to predict the price volatility. The price volatility of SZSE was more likely to be inferred than SSE as D was larger. This is consistent with the previous findings [40]. The Shanghai market was relatively more stochastic than the Shenzhen market (i.e., the Shenzhen market was a little more structured and predictable). This reflects the fact that the Shenzhen market consists of most of the medium- to small-sized companies in China; they are relatively less stable than the large companies. Moreover, the predictability of the price volatility increased when the scale *s* increased.

### 4.2. Daily Data Analysis

**Figure 5:**
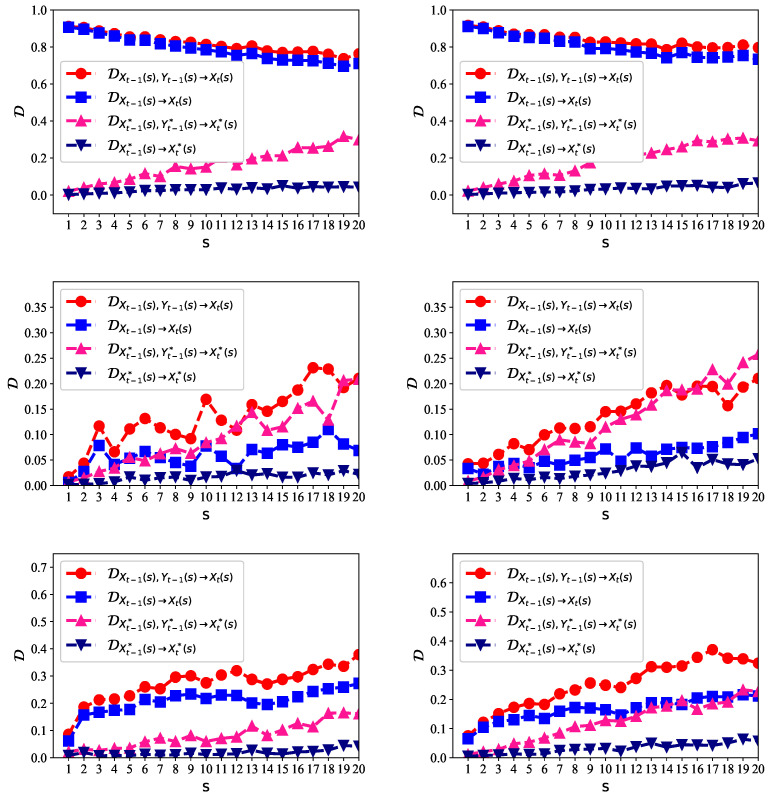
The MED for the daily stock data in the Shanghai and Shenzhen markets. Left panels show the results of the stock price (upper left), logarithmic return (middle left), and price volatility (lower left) for SSE, respectively. Right panels are those for SZSE, respectively. The data related to trading price are given by *X*, and the data related to trading volume are given by *Y*. $X^{*}$ and $Y^{*}$ represent the shuffled data.

We next analyze the predictability of the daily data of SSE and SZSE. The SSE data ranged from 19 December 1990 to 9 October 2018. The SZSE data ranged from 3 April 1991 to 8 October 2018. The correlations between Chinese stock markets and other major stock markets in the world were rather low most of the time. This indicates the fact that Chinese stock markets are relatively independent of the other stock markets, and therefore, we treated the Chinese stock market as an isolated system here. The left panels of Figure 5 show the predictability of the stock price, logarithmic return, and price volatility for SSE, respectively. The right panels show those for SZSE, respectively.

For the non-stationary daily stock prices, the predictability on the upper panels of Figure 5 is high, but meaningless, in the presence of either $X_{t-1}$ alone or $X_{t-1}\&Y_{t-1}$. On the middle panels of Figure 5, the daily logarithmic return is more likely to be predicted given the past values of the logarithmic return and logarithmic change of trading volume than given the past values of the logarithmic return alone: $D_{X_{t-1}\left(s\right),Y_{t-1}\left(s\right)\to X_{t-1}\left(s\right)}$$>D_{X_{t-1}\left(s\right)\to X_{t-1}\left(s\right)}$. However, the shuffled data showed a bit confusing results, as $D_{X_{t-1}\left(s\right),Y_{t-1}\left(s\right)\to X_{t-1}\left(s\right)}$ and $D_{X_{t-1}^{*}\left(s\right),Y_{t-1}^{*}\left(s\right)\to X_{t-1}^{*}\left(s\right)}$ were very close to each other, especially for the Shenzhen market.

We show the results of daily price volatility on the lower panels of Figure 5. The degree of the predictability became larger than that of the daily logarithmic return. Moreover, the MED values were much larger for the daily data than the five-minute data. This indicates that the daily data were more deterministic and predictable than the high-frequency data. The Shanghai market and Shenzhen market showed very similar results.

Our daily data results showed an average degree of predictability. However, they involved times of both high and low volatilities, which implies a change in market behavior. During the times of high volatility (e.g., the 2008 world economic crisis), we found that the degree of predictability increased; while during the times of low volatility, the degree of predictability was much lower. This coincides well with previous studies [40] that the economic crisis can reduce the complexity of stock time series, making the volatility easier to predict.

To be noted, the reasons why we considered only one lag for each variable were two-fold: (i) Suppose that the sampling frequency of the original time series is *f*. In the multiscale analysis, the coarse-graining process, like a low-pass filter, can down sample the time series to f/s. Therefore, for the five-minute high-frequency data, although we used one lag for each variable, we still considered long-distance connections, which were much larger than five minutes. (ii) For most cases, we found that the low-frequency daily data could be approximated by one-order Markov processes. This means that major information could be be exposed by the current daily price and trading volume. Further, past daily data contributed little to predicting the market behavior of the following day, in the presence of the current data.

## 5. Conclusions

In this paper, we introduced a new information-theoretic tool of MED to evaluate the degree of predictability for financial time series. The MED quantifies the contributions of the past values by reducing the uncertainty of the forthcoming values in time series on multiple time scales. For the isolated system, only the past values of the time series alone can be used. However, for the open systems, the past values of the time series and the past values of other time series (which have a close relationship with the underlying time series) can both be utilized. We performed several simulations based on the method, including the logistic map, the Hénon map, and the Lorenz system. All these simulations verified the accuracy and the robustness of our new method. We finally applied the MED method to the analysis of Chinese stock markets. The analysis on the five-minute high-frequency data and daily data of SSE and SZSE revealed that: (i) the logarithmic return had a lower possibility of being predicted than the price volatility; (ii) the trading volume volatility contributed significantly to the prediction of the stock price volatility on multiple time scales; (iii) the daily data were found to have a larger possibility of being predicted than the five-minute high-frequency data.

We note that our new evaluation methods of predictability were based on the assumption of the generalized Markov processes of the underlying time series. However, there still exist many other predicting methods that do not follow such a rule. For example, the k-nearest neighbors (KNN) prediction method [41] and the recurrence quantification analysis (RQA) tool [42] trace out more long-distance past values, so as to match them with the current states. In such a case, our methods would not be applicable any more.
